# 

**Published:** 1877-10-20

**Authors:** 


ORIGINAL AND SELECTED ARTICLES—

Carbuncle—Whitlow and Ingrowing of Nails. By O. E. Newton, M.D. .... 255

Sulphate of Cinchonidia. By C. G. Polk, M.D .... 257

Ammonia Treatment of Rheumatism. By Meriwethe Lewis, A.M., M.D .... 258

The Treatment of Wounds. By Samson Gamgee, F.R.S .... 259

Foreign Bodies in the Ear .... 260

Case of Traumatic Tetanus—Recovery. By G. A. Evans, M.D .... 262

Post Partum Hemorrhage By J. Morrison, M.D .... 268

Case of Membranous Croup—New Method of Treatment—Recovery. By Alexander Fulton, M.D. 265

A Respiratory Brace .... 266

Note on the Treatment of Rupture of the Ligamentum Patellæ .... 266

ABSTRACTS AND GLEANINGS—

Menopause, 267; Amputation of the Arm by Means of the Elastic Ligature, 269; Value of Small Doses of Medicine Frequently Repeated, 270; Vomiting After Debauch, 270; Vomiting in Phthisis, 270; Alimentation of Infants while Suffering from Intestinal Catarrh, 271; Dose and Mode of Administering Nitrate of Silver, 272; Autumnal Diarrhœa, 272; Uses of Hydrate of Chloral, 273; Stringent and Tonic Baths in Summer Complaint of Children, 273; Arsenic in Albumenuria, 273; A Daring Therapeutist, 274; Reduction of Heat in Typhoid Fever, 274; Chronic Malarial Toxemia, 274; Sexual Hygiene, 274; Influence of Artificial Suppression of the Cutaneous Secretion, 275; Insomnia, 275; Belladonna in Constipation, the Result of Paralysis of the Bowels, 275; Treatment of Nasal Catarrh, 275; Post-Partum Hemorrhage, 275; Antiseptic Vinegar, 275; Extraction of Foreign Bodies from the Œsophagus in Children, 276; Pertussis, 276; Podophyllin in the Treatment of Hepatic Colic, 276; Cosmoline, 276; Iodoform in Ear Diseases, 276; Chloral—How Prepared, 276; Poisoning by Salicylic Acid, 276; Sulphophenate of Soda, 276; Diabetes, 276; Treatment of Blennorrhagic Epididymitis with Iodoform Ointment, 277; Ergotin in Metrorrhagia, 277; Idrosis, 277; Fever and Ague, 277; Treatment of Facial Neuralgia, 277; Phosphide of Zinc, 277; Vaseline, 277.

PRACTICAL NOTES AND FORMULÆ—

Tape Worm, 278; Bi-Carbonate of Soda, 278; Yellow Fever, 278; Patent Humbugs, 278; Preserved Summer Squash, 278; Remedy for Sore Eyes, 279; Death from the Want of Salt, 279; Doses for Children, 279; Local Anæsthetic for Gums, 279; To Take Rust out of Steel, 279; Fomula of Indian Cholagogue, 279; Excellent Cough Mixture, 279; Subacute Rheumatic Arthritis, 279; A Good Liniment, 280; Mouth Wash, 280; Anti-Hemorrhagic, 280; On Testicles, and the Procreative Power, 280; Hay Fever, 280; Gargle for Diphtheria, 280; Egg Brandy, 280.

SCIENTIFIC ITEMS—

Telephone, 280; Planet Mars, 280; Locusts, 280.

EDITORIAL AND MISCELLANEOUS—

Special Notice, 281; Extract from Private Letter, 281; A Medical Swindler, 281; Transactions Medical Association of Alabama, April, 1877, 281; Marks of the True Physician, 282; Tobacco, 282; Cinchonida and Cinco-quinine, 282; Negroes and Yellow Fever, 282; The American Pharmaceutical Association, 282; Medical and Surgical Reporter, Philadelphia, 282.

BOOK NOTICES.

Transactions of the Mississippi State Medical Association, 282; Practical Hints on the Selection and Use of the Microscope, 282; Defects of Hearing, and other Evils, 282; The Use of Large Probes, 282; Popular Science Monthly for October, 282; U. S. Dispensatory, 282; Diastasis of the Sternum, 282; Retinitis Albuminuria, 282 ; Transactions of the College of Physicians of Philadelphia, 282.

Special Notice, 253. Plain Talk, but no Offense, 253. Medical Schools, 253. Horse Shoeing, 254. A Misfortune, 254. Book Notices, etc.

